# Life-course exposure to air pollution and the risk of dementia in the Lothian Birth Cohort 1936

**DOI:** 10.1097/EE9.0000000000000355

**Published:** 2024-12-10

**Authors:** Otto-Emil I. Jutila, Donncha Mullin, Massimo Vieno, Samuel Tomlinson, Adele Taylor, Janie Corley, Ian J. Deary, Simon R. Cox, Gergő Baranyi, Jamie Pearce, Michelle Luciano, Ida K. Karlsson, Tom C. Russ

**Affiliations:** a Alzheimer Scotland Dementia Research Centre, University of Edinburgh, Edinburgh, United Kingdom; b Department of Psychology, University of Edinburgh, Edinburgh, United Kingdom; c Lothian Birth Cohorts, University of Edinburgh, Edinburgh, United Kingdom; d Deanary of Molecular, Genetic and Population Health Sciences, University of Edinburgh, Edinburgh, United Kingdom; e Division of Psychiatry, Centre for Clinical Brain Sciences, University of Edinburgh, Edinburgh, United Kingdom; f UK Centre for Ecology & Hydrology (UKCEH), Penicuik, United Kingdom; g Centre for Research on Environment, Society & Health, School of GeoSciences, University of Edinburgh, Edinburgh, United Kingdom; h Centre for Longitudinal Studies, UCL, London, United Kingdom; i Department of Medical Epidemiology and Biostatistics, Karolinska Institute, Stockholm, Sweden.

**Keywords:** Dementia, Air pollution, Alzheimer’s disease, Life-course, PM_2.5_, NO_2_, Sensitive period, Cumulative risk

## Abstract

**Background:**

Air pollution in later life has been associated with dementia; however, limited research has investigated the association between air pollution across the life course, either at specific life periods or cumulatively. The project investigates the association of air pollution with dementia via a life-course epidemiological approach.

**Methods:**

Participants of the Lothian Birth Cohort, born in 1936, provided lifetime residential history in 2014. Participant’s air pollution exposure for time periods 1935, 1950, 1970, 1980, 1990, 2001, and 2007 was modeled using an atmospheric chemistry transport model. Lifetime cumulative exposures were calculated as time-weighted mean exposure. Of 572 participants, 67 developed all-cause dementia [35 with Alzheimer's dementia (AD)] by wave 5 (~82 years). Cox proportional hazards and competing risk models assessed the association between all-cause dementia and AD with particulate matter (diameter of ≤2.5 µm) PM_2.5_ and nitrogen dioxide (NO_2_) exposure at specific life periods and cumulatively. False discovery rate (FDR) correction was applied for multiple testing.

**Results:**

The mean follow-up was 11.26 years. One standard deviation (SD) higher exposure to air pollution in 1935 (PM_2.5_ = 14.03 μg/m^3^, NO_2_ = 5.35 μg/m^3^) was positively linked but not statistically significant to all-cause dementia [PM_2.5_ hazard ratio (HR) = 1.16, 95% confidence interval (CI) = 0.90, 1.49; NO_2_ HR = 1.13, 95% CI = 0.88, 1.47] and AD (PM_2.5_ HR = 1.38, 95% CI = 1.00, 1.91; NO_2_ HR = 1.35, 95% CI = 0.92, 1.99). In the competing risk model, one SD elevated PM_2.5_ exposure (1.12 μg/m^3^) in 1990 was inversely associated with dementia (subdistribution HR = 0.82, 95% CI = 0.67, 0.99) at *P* = 0.034 but not after FDR correction (*P*_FDR_ = 0.442). Higher cumulative PM_2.5_ per one SD was associated with an increased risk of all-cause dementia and AD for all accumulation models except for the early-life model.

**Conclusion:**

The in-utero and early-life exposure to PM_2.5_ and NO_2_ was associated with higher AD and all-cause dementia risk, suggesting a sensitive/critical period. Cumulative exposure to PM_2.5_ across the life course was associated with higher dementia risk. Midlife PM_2.5_ exposure’s negative association with all-cause dementia risk may stem from unaddressed confounders or bias.

**What this study adds:**

Air pollution exposure has been linked to an increased risk of dementia; however, past research exclusively focused on later-life exposure, overlooking the impact of life-course exposure to air pollution. This paper investigated the relationship between air pollution exposure at critical periods and cumulatively across the life course from the in-utero stage until the age of 70 and the subsequent risk of dementia in later life. The findings indicate exposure during certain sensitive/critical periods and cumulatively were associated with dementia.

## Introduction

Ambient air pollution threatens public health, the economy, and the environment.^1^ Over 99% of global inhabitants are exposed to air pollution levels above the WHO standards for air quality.^2^ Air pollution is a complex and heterogeneous mixture of toxic components from anthropogenic and natural sources.^3,4^ Air pollution is composed of gases such as nitrogen oxides (NO_x_), nitrogen dioxide (NO_2_), ozone (O_3_), and particulate matter (PM) ranging from nano to micrometers in size. Most negative health consequences of air pollution have been attributed to fine PM (PM_2.5_, diameter of ≤2.5 µm) and ultrafine particles (diameter of ≤0.1 µm) rather than the coarser PM (PM_10_, diameter less than 10 μm).^5–7^ Air pollution is among the most significant environmental risk factors for human health, with it being the fourth major risk factor for both morbidity and mortality.^8^ The Lancet Commission review concluded that air pollution is one of the 12 key modifiable risk factors for dementia.^9^

Dementia is a group of neurodegenerative disorders that are characterized by a chronic and progressive decline of cognitive function, which impairs independent function for daily activities.^10,11^ However, dementia is not an inevitable part of aging, and modifiable risk factors may prevent or postpone onset.^9,12,13^ Dementia has a long prodromal stage, and risk factors for dementia may exert different effects at distinct life stages or may be cumulative in risk.^14–16^

Recent cohort studies, systematic reviews, and meta-analyses have linked increased long-term air pollution exposure to an elevated risk of dementia.^17–21^ Air pollution exposure is ubiquitous and chronic, impacting large populations, so a slight increase in dementia risk could lead to many cases.^17,22,23^ The impact of air pollution on brain health has been observed at different stages of life.^24,25^ However, studies of the risk of dementia tend to focus on late-life exposure as air pollution data have only been systematically measured for the last couple of decades, and the life-course residential address history of the participants is often lacking.^26^

A recent meta-analysis on air pollution and dementia by Wilker et al^20^ highlighted that varying lengths of exposure time (1–20 years) could result in exposure misclassification and bias results toward the null, as the critical exposure period is unknown. As air pollution is a life-course exposure, it raises the question of when in the life-course does elevated air pollution have a significant, if not the greatest impact on increased risk of dementia (sensitive/critical period of exposure model), or is the risk increased through elevated exposure that accumulates throughout the life-course of the individual (accumulation of risk model).^27,28^ In a prior study of the Lothian Birth Cohort 1936 (LBC1936), PM_2.5_ exposure in-utero was associated with a worse cognitive change from 11 to 70 years when using historical and contemporary air pollution modeled data.^29^ The current study used data from this same cohort and linked air pollution exposure across the lifespan, from in-utero to age 74, to predict the risk of dementia until the age of 85 years. Specifically, we investigated the relationship between air pollution (NO_2_ and PM_2.5_) (1) at various lifetime periods starting in-utero and (2) as accumulated exposure over the life course) and the risk of all-cause dementia and Alzheimer's dementia (AD).

## Methods

### Study population

The data were from the LBC1936, which has been extensively described elsewhere.^30,31^ Participants of this longitudinal cohort study are an ethnically homogenous White sample born in Scotland in 1936, and most took part in the Scottish Mental Survey in 1947, aged approximately 11 years old.^32^ From surviving members of the Scottish Mental Survey living mainly in Edinburgh and the Lothian area, 1,091 men and women were recruited to be part of the LBC1936. The first wave was between 2004 and 2007, when participants were, on average, 70 years old, with follow-up waves repeated approximately every 3 years at an average age of 73 (2007–2010), 76 (2011–2013), 79 (2014–2017), and 82 years (2017–2019).

In 2014, participants were asked to complete a lifetime residential (Life-grid) questionnaire to obtain residential history for the lifetime supported by “flashbulb” memory prompts, selected significant events (e.g., 1984–1985 United Kingdom miners’ strike) that occurred in different life stages of the participants, to reduce recall bias while providing their address histories.^33–35^ Among the 704 participants contacted, 589 participants provided addresses throughout their lives, and 17 participants were excluded because they lived outside of the UK at any time. The remaining 572 participants used for the analysis had, on average, 6.26 [±2.79 standard deviation (SD)] different addresses throughout their lives, ranging from 1 to 18 addresses. The addresses were geocoded to latitude and longitude.^36^ All participants provided informed consent in written form. Ethical approval was granted by the Multi-Centre Research Ethics Committee for Scotland (Wave 1: MREC/01/0/56), the Lothian Research Ethics Committee (Wave 1: LREC/2003/2/29), and the Scotland A Research Ethics Committee (Waves 2–5: 07/MRE00/58).

### Exposure: air pollution

The EMEP4UK (European Monitoring and Evaluation Program Unified Model for the United Kingdom) is an Eulerian atmospheric chemistry transport model (ACTM), which was used to historically model PM_2.5_ and NO_2_ concentrations for the years 1935 (*in utero*), 1950 (~14 years), 1970 (~34 years), 1980 (~44 years), and 1990 (~54 years), which were coupled with contemporary EMEP4UK data for 2001 (~65 years) and 2007 (~71 years). The PM_2.5_ and NO_2_ concentrations were modeled via the EMEP4UK model based on the participants’ residential address history. More details about the EMEP4UK model in terms of setup, configuration, and geographical coverage can be found elsewhere.^37–39^

THE EMPEP4UK model covers the entire of Europe at a grid resolution of 0.5° × 0.5° (50 × 50 km^2^) and was used as a boundary condition with a higher grid resolution of 0.055° × 0.055° (~5 × 5 km^2^) for the UK. In historic air pollution modeling using ACTM, the emissions data can contribute to uncertainty, which was extensively assessed via a sensitivity analysis performed beforehand.^29,40^ Emissions data from Europe were needed for the EMEP4UK model to consider transboundary pollution flows.^41^

The Weather Research and Forecasting Model was the meteorological driver for the EMEP4UK model, the meteorological data from 2014 was used for the 1935 and 1950 emission scenarios, data from 2012 for the emission scenarios of 1970, 1980, and 1990, and the meteorological data for 2001 and 2007 were used for the corresponding emissions scenarios of 2001 and 2007.^42^ The emissions data for 1970, 1980, and 1990 were obtained from the National Atmospheric Emissions Inventory from the official UK inventory.^43^ Emissions data for 1950 were estimated and distributed in the Long-Term Large Scale project.^44^ The 1935 emission data were obtained by scaling the 1950 emissions data based on using the same spatial techniques and research on air pollution-related activity.

This model has been previously used extensively in the UK and worldwide and the modeled air pollutant concentrations have been validated multiple times through comparison with measured concentrations from monitoring networks in the UK.^38,45–47^ The EMEP4UK model has found correlations between the model and measurements for NO_2_ being 0.70–0.76 and PM_2.5_ being 0.65–0.69.^47^ More details on air pollution modeling and using both historical and contemporary air pollution data in health research can be found in past papers.^29,48,49^

The participants’ residential locations were allocated to the closest year that air pollution was modeled for 1935 (the location year 1942 or earlier) was linked to an average of 1.5 (±0.6 SD) addresses during this time period (TP), 1950 (1943–1959) on average had 1.8 (±1.1 SD) addresses, 1970 (1960–1975) on average had 2.8 (±1.3 SD) addresses, 1980 (1976–1985) had on average 1.2 (±0.5 SD) addresses, 1990 (1986–1995) on average had 1.1 (±0.4 SD) addresses, 2001 (1995–2004) on average had 1.1 (±0.25 SD) addresses, or 2007 (2005–2010) had on average 1.0 (±0.2 SD) addresses. Location for air pollution modeling after 2010 was not included to ensure temporality, as dementia was first identified after 2011.

Exposure to air pollution was based on the latitude and longitude of each residential address. As several participants lived at multiple locations within the same TPs, the unweighted mean exposure was calculated for that TP. This meant each participant had six measurements for each type of air pollution to cover the life period of participants.^29,48^ The air pollutants PM_2.5_ and NO_2_ were highly correlated in the early years, and the correlation attenuated in later years (with Pearson correlation coefficients that varied between 0.67 and 0.95) (Appendix Figure S1; http://links.lww.com/EE/A315).

PM_2.5_ and NO_2_ were selected as exposures due to the significant association with increased risk of all-cause dementia and AD in previous studies.^50–53^

### Outcome: dementia

Dementia and subtypes in the LBC1936 were clinically ascertained via consensus from an expert diagnostic review board based on multiple sources of data, including death certificates, hospital discharge data, prescribing data, and review of electronic medical records (EMRs). Diagnosis was based on the International Classification of Disease-Eleventh Edition (ICD-11). In this dataset, the first individual to be diagnosed with dementia was in September 2011. A subset of participants had a home visit by a clinician to perform an extensive clinical assessment when a new dementia diagnosis was self-reported, or memory concerns were raised during LBC1936 wave testing.

The consensus board was comprised of old age psychiatry, geriatric medicine, and neurology clinicians. They agreed upon a diagnosis based on the evidence for probable dementia, possible dementia, or no dementia. Dementia subtypes (e.g., AD) were identified where there was sufficient evidence. Participants were identified as having “probable dementia” if there was an existing dementia diagnosis with no contradicting evidence present on the death certificate, clinical review, or EMRs. The term “probable dementia” is the most certain diagnosis possible based on clinical evidence when a brain biopsy is not available to confirm.^54^ In terms of “possible dementia,” there is evidence of cognitive impairment with no contradictory findings, but it does not meet the ICD-11 criteria. More information about the dementia ascertainment process can be found elsewhere.^55^ All-cause dementia outcome was defined as individuals with probable dementia. In the primary analytic dataset, 67 participants had all-cause dementia, whereas 35 participants had AD.

### Covariates

The covariates were selected from prior literature related to life-course air pollution exposure impact on aging and cognitive decline, and these covariates were added to each model depending on the timing of the covariate.^29,48,53^ Sex (male/female) was consistent for each life-course model. Age was added as the time variable in the Cox proportional hazard models, so was not included as a covariate. Parental’s occupational social class (OSC; dichotomized as professional-managerial [I/II] vs. skilled, partly skilled, and unskilled [III/IV/V]) was included as covariates in the in-utero and early childhood model (1935) and adolescence model (~14 years; 1950). In the adolescence model (~14 years; 1950), childhood smoking (<16 years) was added as a confounder. For models of adulthood and later life (~34 years in 1970 to ~1971 years in 2007), years of education were added, and childhood smoking was replaced with ever-smoking (dichotomized as ever smoker or never smoker) to avoid multicollinearity.

### Statistical analysis

Cox proportional hazard models were used to estimate the hazard ratio (HR) and 95% confidence intervals (CIs) for the association between the specific pollutant with all-cause dementia or AD. Separate models were constructed for exposure in each time band, and various life-course cumulative exposures (Table 1). The underlying time scale was age as days from birth until age at dementia onset, death, or last wave assessment, whichever came first.

**Table 1.** Definitions for sensitive periods and accumulation of risk exposures

Type of life-course model	Exposure
Sensitive/critical periods	Air pollution exposure in-utero and early childhood (1935)
	Air pollution exposure aged ~14 years (1950)
	Air pollution exposure aged ~34 years (1970)
	Air pollution exposure aged ~44 years (1980)
	Air pollution exposure aged ~54 years (1990)
	Air pollution exposure aged ~65 years (2001)
	Air pollution exposure aged ~71 years (2007)
Accumulation of risk	Early life
	(1935 + 1950)
	Early life to young adulthood
	(1935 + 1950 + 1970)
	Early life to mid-adulthood
	(1935 + 1950 + 1970 + 1980)
	Early life to late adulthood
	(1935 + 1950 + 1970 + 1980 + 1990)
	Early life to later life
	(1935 + 1950 + 1970 + 1980 + 1990 + 2001)
	Early life to old age
	(1935 + 1950 + 1970 + 1980 + 1990 + 2001 + 2007)

An unadjusted model was first performed to examine the association between air pollutants with all-cause dementia and AD. Adjusted models were developed with selected covariates dependent on the exposure period. The assumptions for proportional hazards for the Cox regression model were met, based on a Schoenfeld test and visual examination of a plot of scaled Schoenfeld residuals against a log-transformed time.

The two main models used in life-course modeling for all-cause dementia and AD were applied separately for PM_2.5_ and NO_2_, namely the accumulation of risk model and critical/sensitive period model as seen in Table 1.^29^ The sensitive/critical periods approach included seven separate models, which were 1935, 1950, 1970, 1980, 1990, 2001, and 2007. Six accumulation models were constructed to assess the accumulation of air pollution exposure over successively longer periods (Table 1). As the number of years varied for each specific TP of air pollution exposure, the cumulative exposures (*E*) were the time-weighted means calculated as the sum of air pollution exposure during the specific TPs multiplied by the number of years for each the TP (Y_*i*_) divided by the total number of years for the specific TPs (Table 1) with the formula *E* = ΣTP × Y_*i*_/ΣY_*i*_. Similar time-weighted mean exposures have been previously used in studies investigating lifetime exposures to air pollution.^48,56^

The air pollutant exposure was standardized (to mean = 0 and SD = 1) at each TP as the mean air pollutants level and SD varied drastically during the different TPs for PM_2.5_, and the effect per 1 unit would differ substantially.

To address the false discovery rate (FDR) related to the multiple comparison testing (N = 13) of the association between air pollutants at differing TPs and cumulative exposure with all-cause dementia and AD, the Benjamini and Hochberg^57^ procedure was used to obtain a corrected *P* value (*P*_FDR_). This method was used due to being less conservative and more powerful than alternative methods. The multiple imputation was based on all variables used in the adjusted models, and further adjusted models were conducted via chained equations with 15 imputations and predictive mean matching.^58^ The parental OSC was significantly missing and was imputed.

### Sensitivity analysis

For the sensitivity analysis, we included a further adjusted model with additional covariates. All TP models included Apolipoprotein E (*APOE*) ε4 status (dichotomized by the presence or absence of the *APOE* ε4 allele); a fixed variable was identified via genotyping of the polymorphic site *rs429358*).^59^ The childhood IQ score at 11 years old, converted from the Moray House Test during the Scottish Mental Survey 1947 to the standard IQ-type Score, was included for the adolescence and later-life models (~14 years in 1950 to ~71 years in 2007).^60^ Adulthood socioeconomic status was characterized by the highest OSC (I/II vs. III/IV/V) and included as a fixed variable for models of adulthood and later life (~34 years in 1970 to ~1971 years in 2007). In these further adjusted model, multiple imputation was also conducted as these additional variables have significant portion of missing variables. A Fine and Gray competing risk model was used to estimate the proportional subdistribution hazards ratio (_s_HR) to consider the competing risk of death.^61,62^ Air pollution exposure based on only the addresses in the year 1936 was calculated to obtain a more precise estimate of in-utero exposure.

All statistical analyses were conducted using R Statistical software (4.3.0) (R Foundation for Statistical Computing, Vienna, Austria),^63^ and RStudio software (build 386) (Posit, Boston, MA)^64^ using the *survival* (v3.5-5) package for the Cox proportional hazards modeling,^65^ imputation was performed with the *mice* package,^66^ and the *tidycmprsk* (v0.2.0) package for the Fine-Gray model.^67^ Statistical significance was ascertained by a two-sided *P* value (*P* < 0.05).

## Results

**Figure 1.**
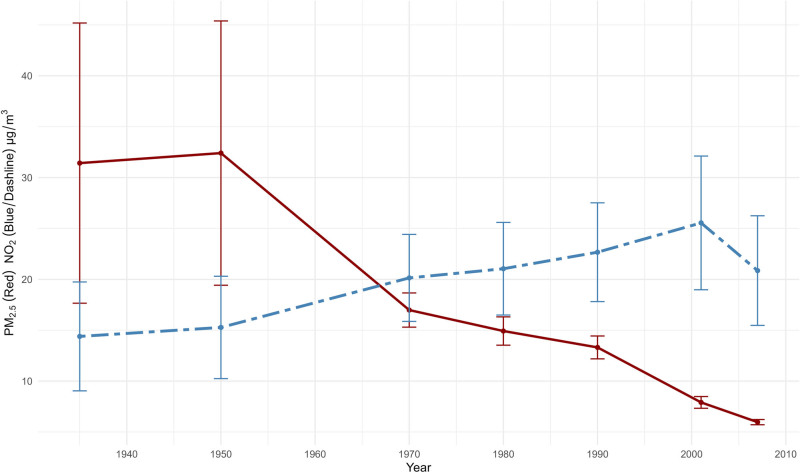
Mean modeled concentration changes for PM_2.5_ and NO_2_ exposure across the individual’s life in the Lothian Birth Cohort 1936. The error bars represent the standard deviation at each time period.

For LBC1936 participants, mean estimated exposure to PM_2.5_ decreased substantially from 1935 to 2007; however, for NO_2_, the mean exposure increased from 1935 to 2001 but decreased in 2007 (Figure 1 and Appendix Table S1; http://links.lww.com/EE/A315). The SDs are larger in earlier years, especially for PM2.5 as the range is larger (Figure 1 and Appendix Table S1; http://links.lww.com/EE/A315). The time-weighted mean accumulative exposure for PM_2.5_ and NO_2_ for each stage are seen in Appendix Table S2; http://links.lww.com/EE/A315.

A summary of baseline characteristics for selected variables is seen in Table 2 for the full sample and separately by all-cause dementia status. Age, sex, ever-smoking status, adult OSC, years of education, and IQ at age 11 were similar between participants with all-cause dementia present or absent (Table 2). Participants with all-cause dementia were more likely to be *APOE*-e4 carriers, be a childhood smoker, and have lower parental OSC when compared with individuals without all-cause dementia (Table 2). The imputed data for baseline characteristics can be seen in Appendix Table S3; http://links.lww.com/EE/A315.

**Table 2.** Baseline characteristics at wave 1 by all-cause dementia status

	Total	All-cause dementia	No all-cause dementia
	N = 572	N = 67	N = 505
Sex (%)			
Male	304 (53)	37 (55)	267 (53)
Female	268 (47)	30 (45)	238 (47)
Age, years			
Mean (±SD)	69.45 (±0.84)	69.49 (±0.86)	69.45 (±0.83)
APOE-e4 status (%)			
One or more e4 alleles	158 (28)	37 (55)	121 (24)
No e4 alleles	384 (67)	26 (39)	358 (71)
Missing	30 (5)	4 (6)	26 (5)
Years of education			
Mean (SD)	10.78 (±1.13)	10.67 (±1.08)	10.80 (±1.13)
Ever Smoker (%)			
Yes	290 (51)	35 (52)	255 (50)
No	282 (49)	32 (48)	250 (50)
Childhood smokers (<16 years) (%)			
Yes	67 (12)	12 (18)	55 (11)
No	505 (88)	55 (82)	450 (89)
Adult occupational social class^a^ (%)			
I and II	338 (59)	41 (61)	297 (59)
III–V	227 (40)	25 (37)	202 (40)
Missing	7 (1)	1 (2)	6 (1)
Parental occupational social class^b^ (%)			
I and II	149 (26)	11 (16)	138 (27)
III–V	389 (68)	50 (75)	339 (67)
Missing	34 (6)	6 (9)	28 (6)
Childhood IQ (aged 11)			
Mean (±SD)	101.59 (±14.96)	101.89 (±14.69)	99.40 (±16.60)
Missing	31 (5%)	2 (3%)	29 (6%)

a Adult occupational social class, based on the Classification of Occupations of Office of Population Censuses and Surveys (1980). I. Professional occupations. II. Managerial and technical occupations. III. Skilled occupations (N) Non-Manual and (M) Manual. IV. Partly skilled occupations. V. Unskilled occupations.

b Parental occupational social class based on the Census 1951 Classification of Occupations of General Register Office (1956). I. Higher managerial, administrative, and professional occupations. II. Intermediate occupations. III. Small employers and own account workers. IV. Lower supervisory and technical occupations. V. Semiroutine and routine occupations.

### Sensitive period models

Elevated exposure to either PM_2.5_ or NO_2_ was not statistically significantly associated with risk of all-cause dementia at any TP or model, except in the competing risk model for PM_2.5_ exposure in 1990 (~54 years old) and inverse risk of all-cause dementia in _s_HR = 0.82 (95% CI = 0.67, 0.99) was significant at the conventional *P* value (*P* = 0.034) but not when corrected by the FDR (*P*_FDR_ = 0.442) (Tables 3 and 4).

**Table 3.** Air pollutant exposures at lifetime periods and association with risk of all-cause dementia

Time periods	SD (μg/m^3^)	Unadjusted model	Adjusted model	Competing risk model
		HR (95% CI)	HR (95% CI)	_S_HR (95% CI)
		N = 572	N = 572	N = 572
PM_2.5_ exposure (per 1 SD)				
1935^a^	14.03	1.17 (0.91, 1.50)	1.18 (0.92, 1.51)^b^	0.95 (0.76, 1.20)^b^
1950^c^	12.84	1.06 (0.86, 1.31)	1.02 (0.82, 1.26)^b^	1.04 (0.82, 1.30)^b^
1970^d^	1.62	1.13 (0.84, 1.52)	1.13 (0.83, 1.53)	1.11 (0.81, 1.53)
1980^d^	1.41	0.80 (0.62, 1.04)	0.80 (0.61, 1.05)	0.84 (0.67, 1.04)
1990^d^	1.12	0.83 (0.64, 1.09)	0.85 (0.65, 1.12)	0.82 (0.67, 0.99)
2001^d^	0.56	0.93 (0.69, 1.25)	0.95 (0.69, 1.30)	0.99 (0.87, 1.13)
2007^d^	0.25	0.94 (0.75, 1.18)	0.94 (0.74, 1.20)	1.02 (0.85, 1.21)
NO_2_ exposure (per 1 SD)				
1935^a^	5.35	1.13 (0.88, 1.45)	1.15 (0.89, 1.49)^b^	0.94 (0.77, 1.16)^b^
1950^c^	5.02	1.05 (0.84, 1.31)	1.01 (0.81, 1.27)^b^	1.01 (0.80, 1.27)^b^
1970^d^	4.27	1.02 (0.78, 1.34)	1.02 (0.77, 1.35)	1.06 (0.78, 1.44)
1980^d^	4.55	0.86 (0.68, 1.10)	0.88 (0.68, 1.13)	0.95 (0.77, 1.19)
1990^d^	4.85	0.91 (0.71, 1.16)	0.92 (0.72, 1.18)	0.94 (0.77, 1.14)
2001^d^	6.57	0.86 (0.67, 1.11)	0.87 (0.68, 1.13)	1.00 (0.83, 1.21)
2007^d^	5.39	0.88 (0.68, 1.13)	0.88 (0.68, 1.14)	1.00 (0.80, 1.26)

a Adjusted model includes sex, parental occupational social class.

b For 34 participants with missing parental occupational social class, this variable was imputed based on 15 imputations.

c Adjusted model includes sex, parental OSC, childhood smoker.

d Adjusted model includes sex, years of education, ever smoker.

**Table 4.** Air pollutant exposures at lifetime periods and association with risk of Alzheimer’s dementia

Time periods	SD (μg/m3)	Unadjusted model	Adjusted model	Competing risk model
		HR (95% CI)	HR (95% CI)	_S_HR (95% CI)
		N = 572	N = 572	N = 572
PM_2.5_ exposure (per 1 SD)				
1935^a^	14.03	1.39 (0.99, 1.94)	1.38 (0.99, 1.93)^b^	1.04 (0.73, 1.48)^b^
1950^c^	12.84	1.06 (0.83, 1.36)	1.00 (0.75, 1.34)^b^	1.13 (0.83, 1.53)^b^
1970^d^	1.62	1.15 (0.75, 1.75)	1.12 (0.71, 1.77)	1.06 (0.60, 1.87)
1980^d^	1.41	0.92 (0.65, 1.32)	1.01 (0.68, 1.51)	1.08 (0.90, 1.28)
1990^d^	1.12	0.94 (0.62, 1.43)	0.99 (0.65, 1.55)	0.95 (0.79, 1.13)
2001^d^	0.56	0.85 (0.56, 1.31)	0.88 (0.54, 1.41)	0.97 (0.84, 1.13)
2007^d^	0.25	0.84 (0.62, 1.14)	0.86 (0.61, 1.23)	0.96 (0.74, 1.24)
NO_2_ exposure (per 1 SD)				
1935^a^	5.35	1.30 (0.90, 1.89)	1.34 (0.91, 1.97)^b^	1.01 (0.73, 1.39)^b^
1950^c^	5.02	1.05 (0.79, 1.39)	0.98 (0.71, 1.37)^b^	1.08 (0.75, 1.56)^b^
1970^d^	4.27	0.93 (0.66, 1.32)	0.86 (0.58, 1.26)	0.89 (0.56, 1.42)
1980^d^	4.55	0.91 (0.64, 1.30)	0.96 (0.64, 1.42)	1.12 (0.80, 1.58)
1990^d^	4.85	0.95 (0.66, 1.36)	0.95 (0.66, 1.38)	0.97 (0.75, 1.25)
2001^d^	6.57	0.78 (0.56, 1.10)	0.80 (0.56, 1.14)	0.97 (0.75, 1.26)
2007^d^	5.39	0.73 (0.52, 1.03)	0.73 (0.51, 1.00)	0.91 (0.68, 1.21)

a Adjusted model includes sex, parental occupational social class.

b For the 34 participants with missing parental occupational social class, this variable was imputed based on 15 imputations.

c Adjusted model includes sex, parental occupational social class, childhood smoker.

d Adjusted model includes sex, years of education, ever smoker.

For the Cox models, the effect estimates were larger for in-utero and early-life exposure (the TP 1935) and went in the inverse direction at later TPs (Tables 3 and 4). The HRs for in-utero exposure to PM_2.5_ and NO_2_ were greater for AD rather than all-cause dementia. However, estimates were generally attenuated toward the null in the competing risk models (Tables 3 and 4). The further adjusted models did not substantially affect the estimates except for in-utero (1935) PM_2.5_ exposure and risk of AD when *APOE* ε4 status was included (HR = 1.38, 95% CI = 1.01, 1.89), which became statistically significant at the conventional *P* value (*P* = 0.040) but not when corrected by the FDR (*P*_FDR_ = 0.523) (Appendix Table S4; http://links.lww.com/EE/A315). The results for the further adjusted model largely remained unchanged after the imputation of missing data (Appendix Table S5; http://links.lww.com/EE/A315). In the sensitivity analysis, air pollution exposure based on only the 1936 address did not differ substantially from in-utero and early-life exposure (TP 1935) (Appendix Table S6; http://links.lww.com/EE/A315).

### Accumulation models

Higher cumulative exposure to PM_2.5_ at any time combination was positively associated, but not to the statistical significance level, with risk of all-cause dementia or AD (Tables 5 and 6). Higher cumulative exposure to NO_2_ was positively associated, but not at the level of statistical significance, with all-cause dementia and AD at most time combinations, but not for 1935–2007 (Tables 5 and 6). For the competing risk models, the effect estimates generally were attenuated toward null compared with the Cox models, however, for PM_2.5_ and AD, the association remained more positive after attenuation, and during 1935–1950 cumulative exposure to PM_2.5_ and NO_2_, the association was strengthened (Tables 5 and 6).

**Table 5.** Accumulation of air pollutant exposures models and association with risk of all-cause dementia

Time periods	SD (μg/m^3^)	Unadjusted model	Adjusted model	Competing risk model
		HR (95% CI)	HR (95% CI)	_S_HR (95% CI)
		N = 572	N = 572	N = 572
PM_2.5_ exposure (per 1 SD)				
1935–1950^a^	12.02	1.11 (0.88, 1.39)	1.06 (0.83, 1.34)^b^	1.01 (0.77, 1.32)^b^
1935–1970^c^	7.01	1.11 (0.89, 1.38)	1.13 (0.91, 1.40)	1.03 (0.81, 1.32)
1935–1980^c^	5.67	1.10 (0.88, 1.37)	1.12 (0.90, 1.39)	1.03 (0.80, 1.31)
1935–1990^c^	4.76	1.09 (0.87, 1.37)	1.11 (0.90, 1.39)	1.02 (0.80, 1.31)
1935–2001^c^	4.23	1.09 (0.87, 1.37)	1.12 (0.90, 1.39)	1.02 (0.80, 1.31)
1935–2007^c^	3.98	1.09 (0.87, 1.37)	1.11 (0.89, 1.39)	1.02 (0.80, 1.31)
NO_2_ exposure (per 1 SD)				
1935–1950^a^	4.74	1.08 (0.85, 1.37)	1.05 (0.82, 1.34)^b^	0.98 (0.77, 1.26)^b^
1935–1970^c^	3.75	1.08 (0.84, 1.39)	1.11 (0.86, 1.43)	1.03 (0.81, 1.30)
1935–1980^c^	3.41	1.00 (0.78, 1.29)	1.04 (0.80, 1.35)	0.99 (0.79, 1.24)
1935–1990^c^	3.29	1.00 (0.78, 1.29)	1.04 (0.80, 1.35)	0.99 (0.79, 1.24)
1935–2001^c^	3.36	0.97 (0.75, 1.25)	1.01 (0.78, 1.31)	0.99 (0.80, 1.23)
1935–2007^c^	3.37	0.96 (0.75, 1.24)	0.99 (0.77, 1.29)	1.00 (0.80, 1.23)

a Adjusted model includes sex, parental occupational social class, childhood smoker.

b For the 34 participants with missing parental occupational social class, this variable was imputed based on 15 imputations

c Adjusted model includes sex, years of education, ever smoker.

**Table 6.** Accumulation of air pollutant exposures models and association with risk of Alzheimer’s dementia

Time periods	SD (μg/m^3^)	Unadjusted model	Adjusted model	Competing risk model
		HR (95% CI)	HR (95% CI)	_S_HR (95% CI)
		N = 572	N = 572	N = 572
PM_2.5_ exposure (per 1 SD)				
1935–1950^a^	12.02	1.14 (0.86, 1.53)	1.07 (0.76, 1.50)^b^	1.13 (0.74, 1.71)^b^
1935–1970^c^	7.01	1.14 (0.86, 1.52)	1.20 (0.91, 1.59)	1.13 (0.78, 1.64)
1935–1980^c^	5.67	1.14 (0.86, 1.52)	1.20 (0.91, 1.60)	1.14 (0.79, 1.64)
1935–1990^c^	4.76	1.14 (0.86, 1.51)	1.20 (0.90, 1.60)	1.13 (0.78, 1.64)
1935–2001^c^	4.23	1.14 (0.86, 1.51)	1.20 (0.91, 1.60)	1.13 (0.78, 1.64)
1935–2007^c^	3.98	1.14 (0.86, 1.51)	1.20 (0.90, 1.60)	1.13 (0.76, 1.64)
NO_2_ exposure (per 1 SD)				
1935–1950^a^	4.74	1.12 (0.81, 1.57)	1.04 (0.71, 1.53)^b^	1.07 (0.69, 1.64)^b^
1935–1970^c^	3.75	1.07 (0.75, 1.51)	1.13 (0.79, 1.62)	1.02 (0.70, 1.49)
1935–1980^c^	3.41	1.02 (0.71, 1.47)	1.09 (0.74, 1.58)	1.03 (0.71, 1.48)
1935–1990^c^	3.29	1.02 (0.71, 1.47)	1.09 (0.74, 1.58)	1.03 (0.71, 1.48)
1935–2001^c^	3.36	0.96 (0.67, 1.38)	1.02 (0.70, 1.49)	1.02 (0.71, 1.45)
1935–2007^c^	3.37	0.94 (0.66, 1.34)	0.99 (0.68, 1.44)	1.01 (0.71, 1.43)

a Adjusted model includes sex, parental occupational social class, childhood smoker.

b For the 34 participants with missing parental occupational social class, this variable was imputed based on 15 imputations.

c Adjusted model includes sex, years of education, ever smoker.

Additional adjustment for adulthood SES, *APOE* ε4 status, and childhood IQ at 11 years old resulted in statistically significant associations for the cumulative PM_2.5_ exposure during 1935–1980, 1935–1990, 1935–2001, and 1935–2007 with the hazard of AD (HR = 1.51, 95% CI = 1.02, 2.21, *P* = 0.04, *P*_FDR_ = 0.48; HR = 1.50, 95% CI = 1.02, 2.21, *P* = 0.04, *P*_FDR_ = 0.50; HR = 1.50, 95% CI = 1.02, 2.20, *P* = 0.04, *P*_FDR_ = 0.52; HR = 1.50, 95% CI = 1.02, 2.20, *P* = 0.04, *P*_FDR_ = 0.53) (Appendix Table S7; http://links.lww.com/EE/A315). The results for the further adjusted model were no longer statistically significant after imputing missing data (Appendix Table S8; http://links.lww.com/EE/A315).

## Discussion

Ours is the first study to assess the risk of air pollution exposure across the lifespan from the early-life stage to the 8th decade of life and the risk of dementia. Whereas the results should be interpreted with caution due to limited power and lack of statistical significance, the findings suggest that in-utero and early childhood (1935) exposure to PM_2.5_ and NO_2_ have a positive association with increased risk of all-cause dementia and AD while air pollution exposure during later periods of life do not, indicating a possible critical/sensitive period. Cumulative exposure to PM_2.5_ was associated with a positive risk of both all-cause dementia and AD, suggesting an accumulative model relationship. In contrast, higher PM_2.5_ exposure at the age of ~44 years (1980) and ~54 years (1990) were associated with the inverse risk of all-cause dementia. However, the CIs often overlap, and results are not statistically significant when adjusted for FDR.

The findings of in-utero and early-life air pollution exposure and risk of AD and all-cause dementia align with another study using the LBC1936 where PM_2.5_ exposure in-utero (1935) was significantly associated with increased cognitive decline from 11 to 70 years.^29^ In a representative Scottish cohort of the same 1936-born population, exposure to PM_2.5_ in childhood (3 years old) was found to be associated with a higher risk of mortality attributed to neurodegenerative disorders in men.^49^

This notion of a sensitive/critical period for risk of dementia fits within the concept of fetal origins of adult disease with prenatal and early postnatal stage considered critical periods for development with significant long-term health consequences.^68,69^ Fetal and childhood periods are susceptible stages to environmental exposures, impacting the developing organs such as the brain and lungs.^70–73^ Specifically, maternal and early-life childhood air pollution exposure can be detrimental to birth outcomes^74,75^ (e.g., smaller head circumference),^76^ neurodevelopment,^77–79^ physiological development, and have long-term health effects.^80–83^ Air pollution may operate through early-life pathways such as body growth,^84^ childhood IQ,^85^ socioeconomic status (SES),^86^ and educational attainment,^87^ which are factors associated with lifetime AD risk with several of these variables were included as confounders in the models for this study.^88–93^ Prenatal and early childhood exposure to air pollutants could, thus, hinder brain development and impact health, which increases the susceptibility to dementia and AD in later life. However, further studies are needed to establish the mediation and moderation between early-life risk factors associated with air pollution and dementia.

The inverse association between PM_2.5_ at the age of ~44 years (1980) and ~54 years (1990) with risk of all-cause dementia is contradictory to both the majority of literature and the underlying biological mechanism. Possible reasons for this unexpected result could be the presence of confounders not adequately adjusted (i.e., socioeconomic status, and urban-rural factors), being a false positive due to the multiple comparisons, or survival bias as higher concentrations in midlife might be due result in mortality before the onset of dementia.^53,94^ Our null findings for air pollution measured at other times over the life course are consistent with studies that found no significant associations between elevated later-life exposure to PM_2.5_ and NO_2_ and higher risk of dementia,^23,95–99^ but not with others, where an association between air pollution, especially PM_2.5_, and risk of dementia has been reported.^17,19,21,100–104^ Typically, studies with larger samples and active ascertainment of dementia were more likely to be statistically significant.^20^

To better model the air pollution and dementia associations, we included a competing risk of death model which showed attenuation of the association of PM_2.5_ and NO_2_ in-utero and early life with all-cause dementia and AD compared with the Cox model. This is in line with a paper by Wu et al^53^, who found attenuation of the association between air pollution and cognitive outcomes in a Fine-Gray model compared with a Cox model, although results remained significant. This could indicate an overestimation of the associations between air pollution and dementia in the literature, resulting from, for example, survival bias and selection effects. However, the Fine-Gray model can underestimate association when covariates in the model are not associated with the primary outcome (all-cause dementia/AD) but are associated with the competing outcome (mortality).^105^ Further assessment of the various competing risk models is needed to assess, which better captures the effect of air pollution on the risk of dementia when considering the risk of mortality.

We found a larger HR for PM_2.5_ with AD when compared with all-cause dementia in line with a large Northern American study and a review.^19^ Indeed, a possible reason for our null findings with all-cause dementia could be heterogeneity in dementia with some subtypes having etiologies that are unconnected to air pollutant exposure, which would shift the hazard to the null. Other studies have included only AD and vascular dementia (VD), or AD, VD, and frontotemporal dementia (FTD) when classifying all-cause dementia^102,106^ but we had insufficient cases to do this for VD and FTD. As AD had a numerically (but not significantly) larger HR than all-cause dementia with air pollution, it may indicate the pathways are mediated by factors other than traditional vascular risk factors,^107^ such as air pollutants directly impacting the brain via inflammation or oxidative stress^108,109^ or other pathways, such as poorer lung function, worse sleep quality, diabetes,^110,111^ and depression.^112^

## Strengths and limitations

This study has a key strength in its novel life-course approach, which used both contemporary and historically modeled air pollution exposure spanning over 8 decades (including air pollution exposure in-utero) with the risk of dementia in later life, allowing the assessment of life-course hypotheses. A novel characteristic of this study is the life-course addresses of participants.^113^ To mitigate recall bias on the retrospective data collection, a life grid approach with “flashbulb” prompts was used, this approach has been previously validated against historical records and shown a high-level accuracy for residential address history in older participants.^34,35^ Dementia and subtype diagnoses in the LBC1936 were clinically ascertained via a validated method including multiple sources of evidence including electronic health records and a home visit, going beyond many other cohort studies that may lack sufficient medical data and underestimate the number of diagnoses, which can bias the results to the null.^20,55^ The accumulation of exposure for PM_2.5_ and NO_2_ was weighted according to length of stay at residential addresses, which could be a more accurate method than unweighted calculations used in prior studies.^29^ However, the optimal method for calculating the accumulation of exposure is not fully known.

The results should be considered with several limitations of the study in mind. Participants provided life-course addresses from only wave 3 (2014), which nearly halved (69/118) the number of cases, significantly reducing the power of the analysis. This later address collection may have been affected by survival bias and selection bias, as the analyzed sample represents a healthier group compared with the entire cohort and the general population. This may partly explain the attenuated effect sizes in the competing risk versus the Cox model. The small size of the cohort and the number of individuals with dementia substantially limited our power to reliably detect small effects of air pollution. Moreover, the limited sample size did not allow for larger models with more covariates to avoid overadjustment and the introduction of colliders, which can introduce residual confounding. However, key life-course cofounders were selected to allow for adequate adjustment, and expanded models were developed in the sensitivity analysis with additional covariates analyzed to mitigate residual confounding. Even with the accurate life grid method for retrospectively collecting address history, there might still be recall bias that increases noise in the measurement. Furthermore, air pollution exposure is based on historical data, with reliability decreasing over time with increasing uncertainty, and the lack of year-specific spatial allocation for air pollution exposure may impact meaningful results. The greatest uncertainties are for emissions during 1935/1950, due to unknown fuel compositions, emission volume verification, and combustion system nature before advancements in technology.^29,47,48,49,114^ The larger positive effect estimates at earlier life stages in this study may be attributable to the significantly higher PM_2.5_ levels exposure at the in-utero stage rather than being a critical/sensitive period; however, the air pollution exposure levels at 1950 are similar to 1935 with comparatively lower effect estimates for all-cause dementia and AD. Our study solely examined residential outdoor air pollution, overlooking complexities in exposure variations at schools, workplaces, commutes, and indoor air pollution. Ideally, such exposure would be included to enhance precision but obtaining requisite data is often impractical.^48^ The LBC1936 participant’s heterogeneity in exposure may be limited with most living in Edinburgh at the cohort’s start, and limited spatial resolution (~5 km × 6 km), especially in the close proximity of urban residing participants all could affect the detection of effects.^48^ The residential address provided for 1936 may not align with the location while in-utero potentially resulting in misclassification of exposure. The parental smoking status for the participants is unknown, which could introduce confounding as maternal smoking while in-utero is associated with lower birth weight, worse neurocognition,^115^ reduced brain development,^116^ and other long-term health problems.^117,118^ The association of maternal smoking with risk of dementia in later life is not certain,^119,120^ but passive smoking during childhood has been associated with risk of dementia.^121^ The LBC1936 sample was ethnically homogenous, which could restrict the study’s generalizability compared with other more diverse populations.

## Implications and conclusion

Previously, air pollution has been viewed as a later life risk factor for dementia when looking at the life-course perspective.^9^ Most literature has focused on later-life exposure and the risk of dementia, often due to the lack of historical air pollution data. This study provides new evidence for understanding the relationship between air pollution and dementia from a life-course perspective, and the potential relevance of in-utero/early-life period and midlife as critical/sensitive TPs. Future research is needed in larger cohorts with increased ethnic diversity and higher resolution air pollution data to confirm whether smaller associations with dementia risk exist that we were underpowered to detect.

## Conflicts of interest statement

The authors declare that they have no conflicts of interest with regard to the content of this report.

## Supplementary Material
