# Supplementary material for: Life-course exposure to air pollution and the risk of dementia in the Lothian Birth Cohort 1936
Source: Environ Epidemiol. 2024 Dec 10;9(1):e355. doi: 10.1097/EE9.0000000000000355 (PMC11634326; doi:10.1097/EE9.0000000000000355)
Supplement: Supplementary file 1 [file ee9-9-e355-s001.docx]

**Supplemental Material:**

Life-course exposure to air pollution and the risk of dementia in the Lothian Birth Cohort 1936

**Figure S1. Correlation between PM_2.5_ and NO_2.5_ at various life periods in the LBC1936**

**Table S1. Mean PM2.5 exposure for participants at each time period**

**Table S2. Time weighted mean for accumulative exposure of PM_2.5_ and NO_2_**

**Table S3. Baseline characteristics at wave 1 by All-Cause Dementia status with imputed data**

**Table S4. Further adjusted model for air pollutant exposures at lifetime periods and association with risk of all-cause dementia model and Alzheimer dementia**

**Table S5. Further adjusted model for air pollutant exposures at lifetime periods and association with risk of all-cause dementia and Alzheimer dementia after multiple imputation**

**Table S6. Air pollutant exposures at 1936 and association with risk of all-cause dementia and Alzheimer dementia**

**Table S7. Further adjusted model for accumulation of air pollutant exposures models and association with risk of all-cause dementia and Alzheimer dementia**

**Table S8. Further adjusted model for accumulation of air pollutant exposures models and association with risk of all-cause dementia and Alzheimer dementia after multiple imputation**

**Appendix**


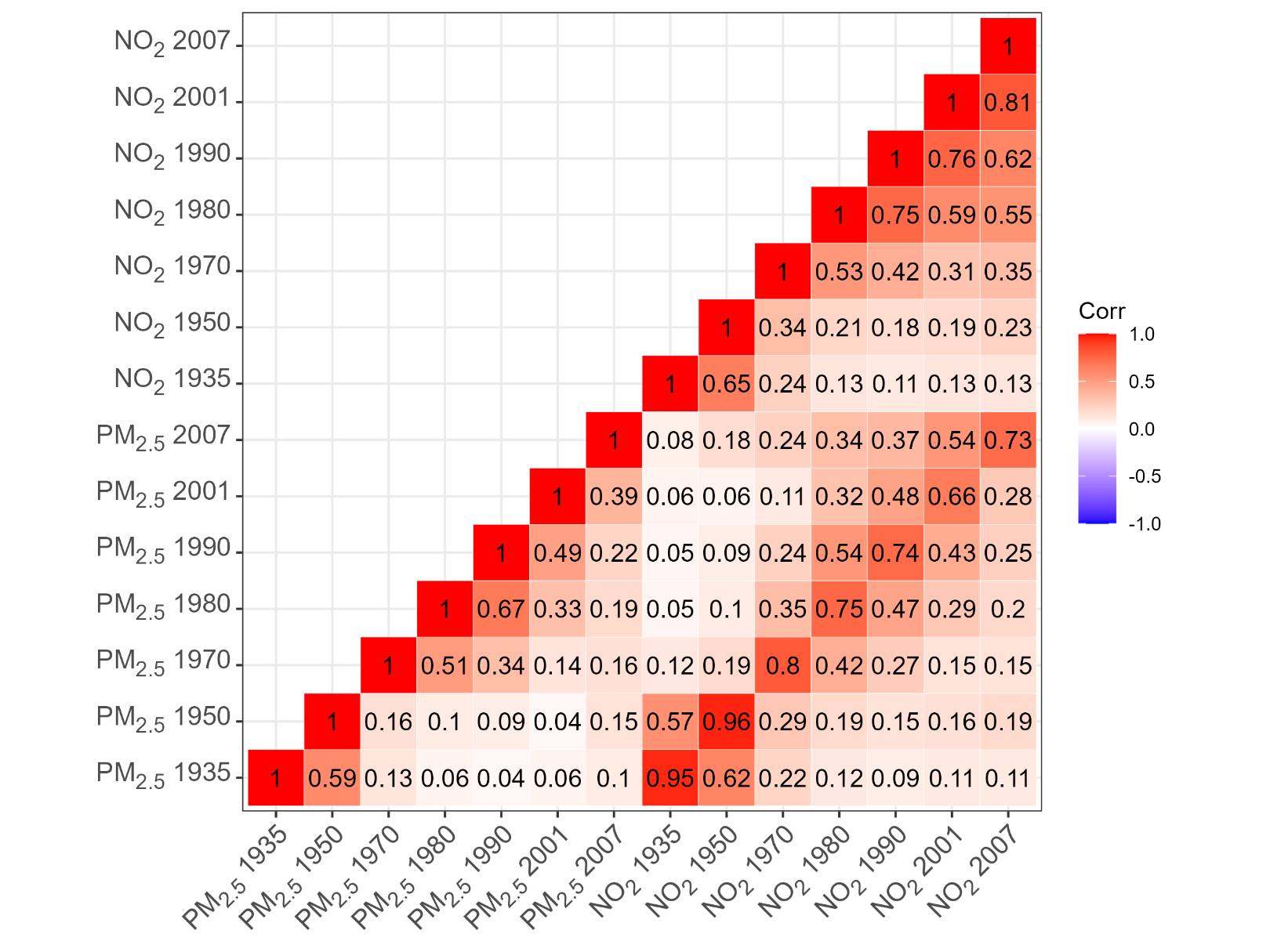


**Figure S1. Correlation between PM_2.5_ and NO_2.5_ at various life periods in the LBC1936**

**Table S1. Mean PM_2.5_ and NO_2_ exposure for participants at each time period**

| **PM_2.5_exposure, (μg/m^3^)** | **Mean** | **Median** | **Range** | **SD** | **IQR*** |
| --- | --- | --- | --- | --- | --- |
| PM_2.5_ 1935 | 31.45 | 35.97 | 5.79-112.30 | 14.03 | 15.39 |
| PM_2.5_ 1950 | 32.22 | 34.29 | 5.96-120.19 | 12.84 | 13.86 |
| PM_2.5_ 1970 | 16.98 | 17.01 | 9.35-25.08 | 1.62 | 0.77 |
| PM_2.5_ 1980 | 14.91 | 14.89 | 7.28-23.98 | 1.41 | 0.58 |
| PM_2.5_ 1990 | 13.30 | 13.21 | 6.67-21.41 | 1.12 | 0.49 |
| PM_2.5_ 2001 | 7.90 | 7.97 | 4.79-15.95 | 0.56 | 0.46 |
| PM_2.5_ 2007 | 5.96 | 5.96 | 4.04-7.77 | 0.25 | 0.27 |
| NO_2_ 1935 | 14.39 | 16.12 | 1.88-34.53 | 5.35 | 7.22 |
| NO_2_ 1950 | 15.72 | 16.40 | 1.93-37.00 | 5.02 | 6.71 |
| NO_2_ 1970 | 20.14 | 21.42 | 3.34-38.96 | 4.27 | 4.67 |
| NO_2_ 1980 | 21.04 | 21.87 | 1.82-50.13 | 4.55 | 2.15 |
| NO_2_ 1990 | 22.67 | 23.49 | 1.42-53.94 | 4.85 | 2.56 |
| NO_2_ 2001 | 25.55 | 28.01 | 3.68-76.04 | 6.57 | 5.45 |
| NO_2_ 2007 | 20.86 | 21.20 | 3.70-29.37 | 5.39 | 4.56 |

*IQR = the 75th percentile–the 25th percentile.

**Table S2. Time weighted mean for accumulative exposure of PM_2.5_ and NO_2_**

| **PM_2.5_ exposure, (μg/m^3^)** | **Mean** | **Median** | **Range** | **SD** | **IQR*** |
| --- | --- | --- | --- | --- | --- |
| Weighted |  |  |  |  |  |
| PM_2.5_ 1935-1950 | 32.14 | 34.21 | 5.94-107.08 | 12.02 | 13.08 |
| PM_2.5_ 1935-1970 | 25.88 | 27.29 | 7.99-68.14 | 7.01 | 7.82 |
| PM_2.5_ 1935-1980 | 23.79 | 24.84 | 7.85-57.69 | 5.67 | 6.24 |
| PM_2.5_ 1935-1990 | 22.09 | 22.93 | 8.72-50.38 | 4.76 | 5.23 |
| PM_2.5_ 1935-2001 | 20.49 | 21.24 | 8.28-45.61 | 4.23 | 4.65 |
| PM_2.5_ 1935-2007 | 19.61 | 20.31 | 8.12-43.21 | 3.98 | 4.38 |
| NO_2_ 1935-1950 | 15.03 | 16.12 | 1.96-34.98 | 4.74 | 6.23 |
| NO_2_ 1935-1970 | 17.08 | 18.06 | 4.39-27.65 | 3.75 | 4.93 |
| NO_2_ 1935-1980 | 17.88 | 18.76 | 4.03-26.48 | 3.41 | 4.62 |
| NO_2_ 1935-1990 | 18.66 | 19.58 | 4.73-26.79 | 3.29 | 4.15 |
| NO_2_ 1935-2001 | 19.45 | 20.27 | 4.63-32.35 | 3.36 | 4.05 |
| NO_2_ 1935-2007 | 19.54 | 20.30 | 5.01-31.52 | 3.37 | 3.88 |

Abbreviations: EL, early life; ELYA, early life to young adulthood; ELMA, early life to mid-adulthood; ELLA, early life to late adulthood; ELLL, early life to later life; ELOA, early life to old age.

*IQR = the 75th percentile–the 25th percentile.

**Table S3. Baseline characteristics at wave 1 by All-Cause Dementia status with imputed data**

|  | Total | All-Cause Dementia Present | All-Cause Dementia Absent |
| --- | --- | --- | --- |
|  | (N=572) | (N=67) | (N=505) |
| **Sex** |  |  |  |
| Male | 304 (53 %) | 37 (55 %) | 267 (53 %) |
| Female | 268 (47 %) | 30 (45 %) | 238 (47 %) |
| **Age, years** |  |  |  |
| Mean (±SD) | 69.45 (±0.84) | 69.49 (±0.86) | 69.45 (±0.83) |
| **APOE-e4 Status** |  |  |  |
| One or more e4 alleles | 168 (29 %) | 39 (58 %) | 126 (25 %) |
| No e4 alleles | 404 (71 %) | 28 (42 %) | 379 (75 %) |
| **Years of Education** |  |  |  |
| Mean (SD) | 10.78 (±1.13) | 10.67 (±1.08) | 10.80 (±1.13) |
| **Ever Smoker** |  |  |  |
| Yes | 290 (51 %) | 35 (52 %) | 255 (50 %) |
| No | 282 (49 %) | 32 (48 %) | 250 (50 %) |
| **Childhood smokers (<16 years)** |  |  |  |
| Yes | 67 (12 %) | 12 (18 %) | 55 (11 %) |
| No | 505 (88 %) | 55 (82 %) | 450 (89 %) |
| **Adult Occupational Social Classᵃ** |  |  |  |
| I and II | 343 (60 %) | 42 (62%) | 303 (60 %) |
| III, IV and V | 229 (40 %) | 25 (38 %) | 202 (40 %) |
| **Parental Occupational Social Class ^b^** |  |  |  |
| I and II | 149 (28 %) | 11 (18 %) | 138 (29 %) |
| III, IV and V | 389 (72 %) | 50 (82 %) | 339 (71 %) |
| **Childhood IQ (Aged 11**) |  |  |  |
| Mean (±SD) | 101.42 (±15.32) | 101.97 (±14.97) | 99.53 (±16.81) |

. ^a^ Adult occupational social class classes, based on the Classification of Occupations of Office of Population Censuses and Surveys (1980). I. Professional occupations, II. Managerial and technical occupations, III. Skilled occupations (N) Non-Manual and (M) Manual, IV. Partly skilled occupations, and V. Unskilled occupations. ^b^ Parental occupational social class based on the Census 1951 Classification of Occupations of General Register Office (1956). I. Higher managerial, administrative, and professional occupations, II. Intermediate occupations, III. Small employers and own account workers, IV. Lower supervisory and technical occupations, and V. Semi-routine and routine occupations

**Table S4. Further adjusted model for air pollutant exposures at lifetime periods and association with risk of all-cause dementia model and Alzheimer dementia**

|  | **All-cause dementia** | **Alzheimer dementia** |
| --- | --- | --- |
| **Time Periods** | HR (95% CI)  (N=61/507) | HR (95% CI)  (N=30/507) |
| PM_2.5_ exposure**,** (per 1 SD) |  |  |
| 1935* | 1.20 (0.94-1.53)^a^ | 1.38 (1.01-1.89)^c^ |
| 1950** | 1.07 (0.84-1.37)^b^ | 1.15 (0.80-1.66)^d^ |
| 1970*** | 1.05 (0.74-1.49) | 0.95 (0.53-1.71) |
| 1980 *** | 0.75 (0.53-1.06) | 1.05 (0.64-1.75) |
| 1990 *** | 0.86 (0.64-1.16) | 1.06 (0.66-1.69) |
| 2001 *** | 0.97 (0.66-1.43) | 0.95 (0.51-1.77) |
| 2007 *** | 1.05 (0.79-1.40) | 0.91 (0.59-1.39) |
| NO_2_ exposure**,** (per 1 SD) |  |  |
| 1935 * | 1.19 (0.91-1.55)^a^ | 1.43 (0.95-2.16)^c^ |
| 1950 ** | 1.03 (0.79-1.35)^b^ | 1.04 (0.67-1.63)^d^ |
| 1970 *** | 0.97 (0.71-1.32) | 0.77 (0.50-1.19) |
| 1980 *** | 0.88 (0.66-1.16) | 0.98 (0.65-1.50) |
| 1990 *** | 0.89 (0.68-1.17) | 0.95 (0.63-1.42) |
| 2001 *** | 0.88 (0.66-1.17) | 0.79 (0.53-1.18) |
| 2007 *** | 0.93 (0.70-1.23) | 0.77 (0.52-1.13) |

* Adjusted model includes sex, parental socioeconomic status, APOE4 status,

** Adjusted model includes sex, parental socioeconomic status, childhood smoker, APOE4 status, IQ at 11 years old

*** Adjusted model includes sex, years of education, ever smoker, APOE4 status, IQ at 11 years old, highest adulthood occupational social class

^a^ The analytic subsample had 512 participants with 57 participants with all-cause dementia due to missingness present with the parental occupational social class variable. ^b^ The analytic subsample had 488 participants with 56 participants with all-cause dementia due to missingness present with the parental occupational social class variable. ^c^ The analytic subsample had 512 participants with 29 participants with Alzheimer’s dementia due to missingness present with the parental occupational social class variable. ^d^ The analytic subsample had 488 participants with 28 participants with Alzheimer’s dementia due to missingness present with the parental occupational social class variable.

**Table S5. Further adjusted model for air pollutant exposures at lifetime periods and association with risk of all-cause dementia and Alzheimer dementia after multiple imputation**

|  | **All-cause dementia** | **Alzheimer dementia** |
| --- | --- | --- |
| **Time Periods** | HR (95% CI)  N=572 | HR (95% CI)  N=572 |
| PM_2.5_ exposure**,** (per 1 SD) |  |  |
| 1935* | 1.20 (0.95-1.52)^a^ | 1.41 (1.03-1.92)^a^ |
| 1950** | 1.10 (0.88-1.38)^b^ | 1.20 (0.87-1.67)^b^ |
| 1970*** | 1.15 (0.84-1.58)^c^ | 1.14 (0.69-1.88)^c^ |
| 1980 *** | 0.82 (0.62-1.10)^c^ | 1.03 (0.67-1.58)^c^ |
| 1990 *** | 0.88 (0.68-1.14)^c^ | 0.98 (0.66-1.45)^c^ |
| 2001 *** | 0.98 (0.70-1.38)^c^ | 0.90 (0.53-1.50)^c^ |
| 2007 *** | 0.99 (0.75-1.31)^c^ | 0.93 (0.61-1.40)^c^ |
| NO_2_ exposure**,** (per 1 SD) |  |  |
| 1935 * | 1.20 (0.93-1.54)^a^ | 1.46 (0.98-2.18)^a^ |
| 1950 ** | 1.09 (0.86-1.39)^b^ | 1.20 (0.81-1.76)^b^ |
| 1970 *** | 1.04 (0.78-1.40)^c^ | 0.89 (0.58-1.37)^c^ |
| 1980 *** | 0.90 (0.69-1.16)^c^ | 1.00 (0.67-1.50)^c^ |
| 1990 *** | 0.91 (0.71-1.17)^c^ | 0.93 (0.64-1.34)^c^ |
| 2001 *** | 0.88 (0.67-1.14)^c^ | 0.79 (0.55-1.15)^c^ |
| 2007 *** | 0.90 (0.69-1.17)^c^ | 0.78 (0.53-1.14)^c^ |

* Adjusted model includes sex, parental occupational social class. APOE4 status ** Adjusted model includes sex, parental socioeconomic status, childhood smoker, APOE4 status, IQ at 11 years old. *** Adjusted model includes sex, years of education, ever smoker, APOE4 status, IQ at 11 years old, highest adulthood occupational social class. ^a^ For 34 participants with missing parental occupational social class and 30 participants with missing APOE-e4 status, had these variables imputed based on 15 imputations. ^b^ For 34 participants with missing parental occupational social class, 30 participants with missing APOE-e4 status, and 31 participants with missing childhood IQ at aged 11, had these variables imputed based on 15 imputations. ^c^ For 30 participants with missing APOE-e4 status, 31 participants with missing childhood IQ at aged 11, and 7 participants with missing adult occupational social class, had these variables imputed based on 15 imputations.

**Table S6. Air pollutant exposures at 1936 and association with risk of all-cause dementia and Alzheimer dementia**

|  | **All-cause dementia** | **Alzheimer dementia** |
| --- | --- | --- |
| **Time Periods** | HR (95% CI) | HR (95% CI) |
| PM_2.5_ exposure**,** (per 1 SD) |  |  |
| 1936* | 1.20 (0.95-1.52)^a^ | 1.31 (0.97-1.79)^a^ |
| NO_2_ exposure**,** (per 1 SD) |  |  |
| 1936 * | 1.12 (0.92-1.55)^a^ | 1.31 (0.88-1.95)^a^ |

* Adjusted model includes sex, parental occupational social class. ^a^ For the 34 participants with missing parental occupational social class, this variable was imputed based on 15 imputations

**Table S7. Further adjusted model for accumulation of air pollutant exposures models and association with risk of all-cause dementia and Alzheimer dementia**

|  | All-cause dementia | Alzheimer dementia |
| --- | --- | --- |
| **Time Periods** | HR (95% CI)  (N= 61/507) | HR (95% CI)  (N= 30/507) |
| PM_2.5_ exposure**,** (per 1 SD) |  |  |
| 1935-1950* | 1.14 (0.87-1.50)^a^ | 1.32 (0.86-2.04)^b^ |
| 1935-1970** | 1.26 (0.99-1.61) | 1.32 (0.86-2.04) |
| 1935-1980** | 1.25 (0.98-1.60) | 1.51 (1.02-2.21) |
| 1935-1990** | 1.25 (0.98-1.59) | 1.50 (1.02-2.21) |
| 1935-2001** | 1.25 (0.98-1.59) | 1.50 (1.02-2.20) |
| 1935-2007** | 1.25 (0.98-1.59) | 1.50 (1.02-2.20) |
| NO_2_ exposure**,** (per 1 SD) |  |  |
| 1935-1950* | 1.09 (0.82-1.46)^a^ | 1.20 (0.72-2.00)^b^ |
| 1935-1970** | 1.21 (0.91-1.62) | 1.27 (0.79-2.03) |
| 1935-1980** | 1.10 (0.83-1.46) | 1.15 (0.74-1.77) |
| 1935-1990** | 1.10 (0.83-1.46) | 1.15 (0.74-1.77) |
| 1935-2001** | 1.06 (0.80-1.41) | 1.07 (0.70-1.62) |
| 1935-2007** | 1.05 (0.79-1.39) | 1.03 (0.69-1.56) |

* Adjusted model includes sex, parental socioeconomic status, childhood smoker, APOE4 status, IQ at 11 years old.** Adjusted model includes sex, years of education, ever smoker, APOE4 status, IQ at 11 years old, highest adulthood occupational social class. ^a^ The analytic subsample had 488 participants with 56 participants with all-cause dementia due to missingness present with the parental occupational social class variable. ^b^The analytic subsample had 488 participants with 28 participants with Alzheimer’s dementia due to missingness present with the parental occupational social class variable.

**Table S8. Further adjusted model for accumulation of air pollutant exposures models and association with risk of all-cause dementia and Alzheimer dementia after multiple imputation**

|  | All-cause dementia | Alzheimer dementia |
| --- | --- | --- |
| **Time Periods** | HR (95% CI) | HR (95% CI) |
| PM_2.5_ exposure**,** (per 1 SD) |  |  |
| 1935-1950* | 1.15 (0.90-1.47)^a^ | 1.34 (0.92-1.95)^a^ |
| 1935-1970** | 1.22 (0.97-1.53)^b^ | 1.39 (0.97-1.98)^b^ |
| 1935-1980** | 1.21 (0.96-1.52)^b^ | 1.39 (0.97-1.98)^b^ |
| 1935-1990** | 1.20 (0.95-1.51)^b^ | 1.39 (0.97-1.98)^b^ |
| 1935-2001** | 1.20 (0.95-1.52)^b^ | 1.39 (0.97-1.98)^b^ |
| 1935-2007** | 1.20 (0.95-1.52)^b^ | 1.39 (0.97-1.98)^b^ |
| NO_2_ exposure**,** (per 1 SD) |  |  |
| 1935-1950* | 1.14 (0.88-1.47)^a^ | 1.31 (0.84-2.04)^a^ |
| 1935-1970** | 1.21 (0.92-1.57)^b^ | 1.25 (0.81-1.94)^b^ |
| 1935-1980** | 1.11 (0.84-1.45)^b^ | 1.15 (0.76-1.75)^b^ |
| 1935-1990** | 1.11 (0.84-1.45)^b^ | 1.15 (0.76-1.75)^b^ |
| 1935-2001** | 1.06 (0.81-1.39)^b^ | 1.06 (0.71-1.59)^b^ |
| 1935-2007** | 1.04 (0.80-1.37)^b^ | 1.03 (0.69-1.54)^b^ |

* Adjusted model includes sex, parental socioeconomic status, childhood smoker, APOE4 status, IQ at 11 years old.** Adjusted model includes sex, years of education, ever smoker, APOE4 status, IQ at 11 years old, highest adulthood occupational social class. ^a^ For 34 participants with missing parental occupational social class, 30 participants with missing APOE-e4 status, and 31 participants with missing childhood IQ at aged 11, had these variables imputed based on 15 imputations. ^b^ For 30 participants with missing APOE-e4 status, 31 participants with missing childhood IQ at aged 11, and 7 participants with missing adult occupational social class, had these variables imputed based on 15 imputations.
